# Diabetes mellitus and hypertension care in Bidur Municipality, Nuwakot District, Nepal: A cross-sectional study

**DOI:** 10.1097/MD.0000000000044303

**Published:** 2025-09-05

**Authors:** Gambhir Shrestha, Deepak Raj Joshi, Pranil Man Singh Pradhan, Sushan Man Shrestha, Namrata Karki, Prem Basel, Smriti Pant, Tanweer Ahmad Mikrani, Khem Bahadur Karki

**Affiliations:** a Department of Community Medicine and Public Health, Maharajgunj Medical Campus, Institute of Medicine, Tribhuvan University, Kathmandu, Nepal; b Central Department of Public Health, Institute of Medicine, Tribhuvan University, Kathmandu, Nepal.

**Keywords:** care, diabetes, hypertension, Nepal

## Abstract

Chronic diseases, such as diabetes mellitus (DM) and hypertension (HTN), are growing public health concerns globally, with notably low management rates in low- and middle-income countries. This study aimed to assess the care-related outcomes of DM and HTN in Bidur Municipality, Nepal. This cross-sectional study was conducted in Bidur Municipality in Nuwakot District of Nepal. A convenient sampling method was used to select 3183 households, and 1 preferably the head of the household ≥18 years, was interviewed. Data were collected using a Nepali-translated questionnaire consisting of sociodemographic characteristics, health-seeking behavior, insurance, substance use, family history, and screening, diagnosis, and treatment for both DM and HTN. Multiple logistic regression was applied to examine associations between care-related outcomes and explanatory variables. Among 3183 participants, 43% had ever been screened for DM and 65% for HTN. Of those screened, 68% had DM screening, and 82% had blood pressure checks within the past 6 months. The prevalence of DM and HTN was 18.7% and 26.1%, respectively. Among those diagnosed, 74.3% with DM and 76% with HTN were receiving treatment, with continuation rates of 98.4% and 97%, respectively. DM screening was significantly associated with age, ethnicity, family history, and insurance; diagnosis with age, ethnicity, and family history; and treatment adherence with family history. HTN screening was linked to age, family size, and family history; diagnosis to older age, family type, and family history; and adherence to treatment to age, ethnicity, family history, and insurance. Screening rates for DM and HTN were higher in Bidur Municipality compared to that of the national data. However, disparities persist, particularly among certain ethnic groups and uninsured individuals, who have lower screening rates. Older age and a positive family history consistently predicted screening, diagnosis, and treatment adherence for both conditions.

## 1. Introduction

Diabetes Mellitus (DM) and hypertension (HTN) are emerging chronic diseases posing public health challenges in the world. Evidence reports that a significant number of people with DM and HTN reside in low and middle-income countries, where the management of these diseases is notably low.^[1,2]^

According to a meta-analysis report, the pooled prevalence of DM, awareness, treatment, and control in Nepal was 8.5% (95% confidence interval [CI]: 6.9–10.4%), 52.7% (95% CI: 41.7–63.4%), 45.3% (95% CI: 31.6–59.8%), and 36.7% (95% CI 21.3–53.3%) respectively.^[3]^ Similarly another meta-analysis reports the pooled prevalence of HTN, awareness, treatment, and control in Nepal was 32% (95% CI: 23–40%), 50% (95% CI: 30–69%), 27% (95% CI: 19–34%), and 38% (95% CI: 28–48%), respectively.^[4]^ Both meta-analyses have reported a high prevalence of DM and HTN, and their relatively low awareness, treatment, and control in Nepal.^[3,4]^

It is essential to understand the various care-related outcomes of care, including screening rates and treatment adherence, for these diseases to identify strategies to reduce their burden and prevent complications. This will also help target intervention programs and improve the delivery of health services. This study aims to find out the proportion and predictors of different care-related outcomes of DM and HTN in Bidur Municipality, Nuwakot District, Nepal.

## 2. Materials and methods

### 2.1. Study setting

This was a cross-sectional study conducted in Bidur Municipality, located in Nuwakot District, Nepal, between January 19, 2024, and February 18, 2024. This was a part of a larger Community Health Diagnosis Program in the Municipality.

### 2.2. Sampling method and sample size

Out of 13 wards in Bidur Municipality, 10 were chosen purposively in this study. The total households in Bidur Municipality as per the 2021 census was 15,234.^[5]^ A convenient sampling approach was used, selecting approximately 25% of the total households in each selected ward as a sample. We surveyed 3183 households in this study. In each household, 1 person was interviewed, preferably the head of the household. In the absence of the head of the household, the eldest member was selected for the study.

### 2.3. Inclusion criteria

This study included adults aged 18 years and older residing in Bidur Municipality.

### 2.4. Data collection

This study was a part of the Community Health Diagnosis field program of MBBS first-year students. They were provided with in-depth 5-day training and orientation on the CHD questionnaire before data collection for tool validity. The tool was pretested in the households of the Tokha Municipality. The data collection was done house-to-house under the direct supervision of the faculty of the Department of Community Medicine and Public Health.

The Nepali-translated questionnaire was administered using a face-to-face interview technique. The CHD questionnaire consisted of various epidemiological and social determinants of health. In this study, we assessed sociodemographic characteristics, health-seeking behavior, insurance, substance use, family history, and different care-related outcomes of care for DM and HTN.

The data were collected using the Kobo toolbox, which is a freely available data collection and management software that supports both Android and iOS platforms. All the collected data was stored in a cloud server of the Kobo toolbox. The quality of data and any errors in data entry were monitored by the research team of the Department of Community Medicine and Public Health.

### 2.5. Independent variables

The sociodemographic characteristics included gender (male, female), age group (18–40, 41–60, over 60 years), marital status (married or living together and divorced, widowed, separated, or single), caste (Brahmin/Chhetri, Janajati, others), Religion (Hindu, others), educational status (illiterate, up to primary level, secondary and above), occupation (unemployed/retired, professionals/business, labor or working in daily wage, agriculture, students and homemaker). Later, it was dichotomized into income non-generating occupation and income-generating occupation, family size (1–3 members, 4–5 members, more than 6 members), and family type (nuclear, joint). Health-seeking behavior was assessed by the first place where they visit for health problems (government, private, others) and insurance status (insured, not insured).

Alcohol use was defined as any self-report of alcohol consumption in the past 30 days. Tobacco use was defined as self-report of use of tobacco in any form (smoking and/or smokeless tobacco) in the past 30 days. Family history of DM and HTN was assessed through self-reporting of these conditions in the family.

### 2.6. Outcome variables

Different care-related outcomes for DM and HTN were constructed with 4 stages: Ever screened among the study population, prior diagnosis among those screened, among those diagnosed, ever used medication for that condition, and among those with prior medication, the proportion currently using medication. For respondents ever on treatment, they were still considered currently on treatment if they were engaged in care and the health care providers advised them not to take medicine as their condition is under control.

DM and HTN were defined as a previous diagnosis of DM and HTN by a health care provider or ever having received treatment for these conditions. The status of screening was assessed by ever checked or tested their blood pressure or blood glucose level.

### 2.7. Statistical analysis

All the collected data were stored in a cloud server of the Kobo toolbox. The quality of data and any errors in data entry were monitored by the Department of Community Medicine and Public Health. Constant feedback was given to the students in the field regarding the same. The collected data was extracted as an MS Excel file and exported to Statistical Package for Social Sciences version 20. Data were presented in the form of tables and graphs. Frequencies and percentages were calculated for descriptive statistics. We conducted bivariate analysis for each of the explanatory variables for the care-related outcomes of DM and HTN. The variables with a *P*-value <.2 in the bivariate analysis were considered for multiple logistic regression. Adjusted odds ratios (AOR) and 95% CI were calculated to present the strength of association of screened, diagnosed, and currently diagnosed DM and HTN with sociodemographic and other related variables.

### 2.8. Ethical considerations

The Institutional Review Committee of the Institute of Medicine, Tribhuvan University approved this study. Written informed consent was obtained from the participants prior to the collection of the data.

## 3. Results

### 3.1. Sociodemographic characteristics

Among 3183 households surveyed, the mean age of the participants was 43.4 (SD = 15.5) years. The commonest age group was 18 to 40 years, followed by 41 to 60 years. The male-to-female ratio was 0.64. About 57.3% of the participants were married and living together. More than half of them were Brahmin/ Chhetri, followed by Janajati. The majority (87%) were Hindu by religion. Almost half (49%) had a family size of 4 to 5 in number, followed by 1 to 3 (27%) and 6 or more (24%). More than half (58%) were living in a nuclear family type. About 38% of the participants had completed secondary and above educational level, followed by up to primary level (31%), and illiterate (31%). About 38% of the participants were unemployed/retired, followed by professionals (28%) and homemakers (15%; Table 1).

**Table 1:** Sociodemographic characteristics of the participants (n = 3183).

Characteristics	Categories	Number (n)	Percentage
Age (yr)	Mean (SD)	43.4 (15.5)	
	18–40	1613	50.7
	41–60	1064	33.4
	>60	506	15.9
Gender	Male	1238	38.9
	Female	1945	61.1
Marital status	Single/divorced/widow/separated	1360	42.7
	Married	1823	57.3
Caste	Brahmin/Chhetri	1632	51.3
	Janajati	1042	32.7
	Others	509	16.0
Religion	Hindu	2771	87.1
	Others	412	12.9
Family type	Nuclear	1835	57.7
	Joint	1348	42.3
Family size	1–3	848	26.6
	4–5	1564	49.1
	≥6	771	24.2
Education	Illiterate	981	30.8
	Up to primary level	1000	31.4
	Secondary and above	1202	37.8
Occupation	Unemployed/retired	1216	38.2
	Agriculture	296	9.3
	Professionals/business	898	28.2
	Student	93	2.9
	Labor/daily wage worker	139	4.4
	Home maker	476	15.0
	Others	65	2.0

SD = standard deviation.

### 3.2. Substance use and health-seeking behavior

A total of 22% and 16% of the participants were current tobacco users in any form and current alcohol consumers, respectively. A majority (76%) seek health care first at government facilities. Only 43% had health insurance (Table 2).

**Table 2:** Substance use and health-seeking behavior of the participants (n = 3183).

Characteristics	Categories	Number (n)	Percentage
Currently using tobacco	Yes	700	22.0
	No	2483	78.0
Alcohol consumption	Yes	516	16.2
	No	2667	83.8
Seek care first at	Government	2405	75.6
	Private	726	22.8
	Others	52	1.6
Health insurance	Insured	1369	43.0
	Not insured	1814	57.0

### 3.3. Care-related outcomes of diabetes

**Figure 1.**
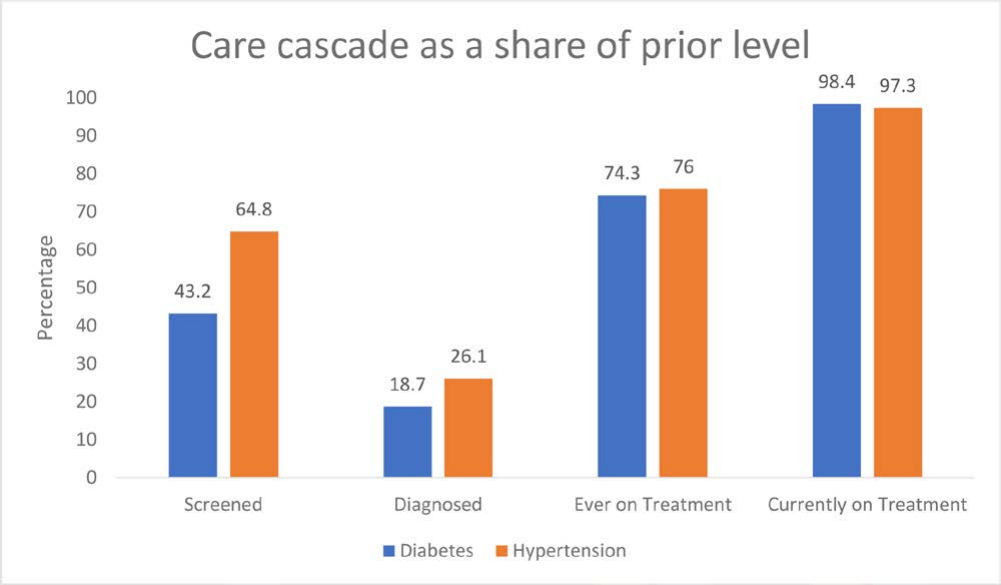
Care-related outcomes of diabetes and hypertension among the participants (n = 3183).

Only 43% of participants had ever screened for DM. Among those screened, 68% had their last screening for DM within 6 months. About 17% of the participants had a positive family history of DM (Table 3). The prevalence of DM was found to be 18.7% (95% CI: 16.7–20.9). Among those diagnosed with DM, 74.3% have been in treatment, and 98.4% continued it (Fig. 1).

**Table 3:** Diabetes and hypertension screening practices and family history among the participants (n = 3183).

Characteristics	Categories	Diabetes (n)	Percentage	Hypertension (n)	Percentage
Family history	Yes	543	17.1	1016	31.9
Screened	Yes	1375	43.2	2063	64.8
Last tested among those screened	Within 3 mo	698	50.8	1430	69.3
	3–6 mo	232	16.9	257	12.5
	6–12 mo	145	10.5	144	7.0
	More than 12 mo	300	21.8	232	11.2
Diagnosed among those screened	Yes	257	18.7	538	26.1

### 3.4. Care-related outcomes of hypertension

Only 65% of participants had ever been screened for HTN. Among those screened, 82% had their last screening for HTN within 6 months. About 17% of the participants had a positive family history of HTN. The prevalence of HTN among those ever screened was 26.1% (95% CI: 24.2–28.1). Among those diagnosed for HTN, 76% have been in treatment and 97% have continued it.

### 3.5. Predictors of diabetes care

Age was positively associated with increasing screening rate of DM (AOR = 1.01; 95% CI: 1.01–1.02). Participants from Janajati (AOR = 0.79; 95% CI: 0.64–0.97) and other caste groups (AOR = 0.45; 95% CI: 0.35–0.59) exhibited a lower screening rate compared to Brahmin/Chhetri individuals. A positive family history of DM was associated with 3.9 times higher odds of undergoing DM screening compared to those without such a history (AOR = 3.87; 95% CI: 3.06–4.89). Insured participants had 1.5 times higher odds of being screened for DM compared to their noninsured counterparts (AOR = 1.5; 95% CI: 1.27–1.80).

Regarding DM diagnosis, age was significantly associated with increased likelihood of diagnosis (AOR = 1.03; 95% CI: 1.02–1.05). Individuals from Janajati and other caste groups showed a higher prevalence of DM compared to Brahmin/Chettri, though only the other caste category was statistically significant (AOR = 1.93; 95% CI: 1.03–3.63). A positive family history was associated with 71 times higher odds of being diagnosed with DM (AOR = 71.6; 95% CI: 41.5–123.4).

For adherence to DM treatment, only a positive family history of DM emerged as a significant predictor (AOR = 8.9; 95% CI: 2.75–29.1; Table 4).

**Table 4:** Predictors of care-related outcomes of diabetes.

Characteristics	Ever screened		Diagnosed		Currently on treatment	
	AOR (95% CI)	*P*-value	AOR (95% CI)	*P*-value	AOR (95% CI)	*P*-value
Age	1.01 (1.01–1.02)	<.001	1.03 (1.02–1.05)	<.001	1.01 (0.99–1.03)	.427
Gender (Female = 1)	1.15 (0.96–1.37)	.133	1.18 (0.81–1.73)	.394		
Marital status (Married = 1)			1.23 (0.86–1.78)	.261		
Caste (Brahmin/Chhetri = 1)						
Janajati	0.79 (0.64–0.97)	.022	1.41 (0.88–2.26)	.154	1.54 (0.76–3.13)	.233
Others	0.45 (0.35–0.59)	<.001	1.93 (1.03–3.63)	.040	1.87 (0.69–5.06)	.218
Religion (Hindu = 1)	1.03 (0.78–1.36)	.854	0.66 (0.33–1.30)	.228		
Family type (Nuclear = 1)			0.85 (0.55–1.32)	.469		
Family size (1–3 = 1)						
4–5			0.95 (0.57–1.57)	.835		
6 and more			0.69 (0.38–1.25)	.221		
Education (Illiterate = 1)						
Up to primary level	1.14 (0.92–1.41)	.232				
Secondary and above	0.92 (0.75–1.13)	.436				
Family history of DM (No = 1)	3.87 (3.06–4.89)	<.001	71.60 (41.53–123.44)	<.001	8.94 (2.74–29.16)	<.001
Insurance (Not insured = 1)	1.51 (1.27–1.80)	<.001			1.52 (0.84–2.76)	.168

1 indicates reference group.

AOR = adjusted odds ratio, CI = confidence interval, DM = diabetes mellitus.

### 3.6. Predictors of hypertension care

Older age was positively associated with HTN screening (AOR = 1.02; 95% CI: 1.01–1.03). Individuals from “other” caste groups had lower odds of being screened compared to Brahmin/Chettri (AOR = 0.53; 95% CI: 0.39–0.72). Larger family sizes were associated with higher odds of screening compared to smaller family sizes. Those from families of 4 to 5 members had 1.44 times higher odds (AOR = 1.44; 95% CI: 1.11–1.87) and those from families of 6 or more members had 1.58 times higher odds (AOR = 1.58; 95% CI: 1.10–2.27)for screening than those of smaller families. A positive family history of HTN was associated with increased likelihood of being screened compared to those without such a history (AOR = 2.15; 95% CI: 1.67–2.76).

For being diagnosed with HTN, older age was significantly associated with HTN diagnosis, with an AOR of 1.06 (95% CI: 1.05–1.07). Individuals from joint families had lower odds of HTN diagnosis compared to those from nuclear families (AOR = 0.61; 95% CI: 0.44–0.85). A positive family history of HTN was associated with HTN diagnosis (AOR = 82.56; 95% CI: 50.57–134.79).

Regarding adherence to HTN treatment, older age was associated with higher odds of treatment adherence, with an AOR of 1.05 (95% CI: 1.03–1.06). Individuals from the Janajati caste had lower odds of adherence compared to Brahmin/Chhetri caste groups, with an AOR of 0.51 (95% CI: 0.31–0.84). A positive family history of HTN was associated with increased adherence to treatment, with an AOR of 6.29 (95% CI: 2.20–17.98). Insured individuals had higher odds of adhering to HTN treatment compared to noninsured individuals, with an AOR of 2.18 (95% CI: 1.39–3.41; Table 5).

**Table 5:** Predictors of care-related outcomes of hypertension.

Characteristics	Ever screened		Diagnosed		Currently on treatment	
	AOR (95% CI)	*P*-value	AOR (95% CI)	*P*-value	AOR (95% CI)	*P*-value
Age	1.02 (1.01–1.03)	<.001	1.06 (1.05–1.07)	<.001	1.05 (1.03–1.06)	<.001
Gender (Female = 1)			1.22 (0.91–1.64)	.175	1.41 (0.89–2.22)	.143
Marital status (Married = 1)	0.89 (0.72–1.10)	.286	1.24 (0.94–1.65)	.131		
Caste (Brahmin/Chhetri = 1)						
Janajati	0.89 (0.69–1.13)	.326			0.51 (0.31–0.84)	.008
Others	0.53 (0.39–0.72)	.000			1.31 (0.65–2.63)	.456
Religion (Hindu = 1)					0.68 (0.35–1.33)	.257
Family type (Nuclear = 1)	0.89 (0.68–1.15)	.370	0.61 (0.44–0.85)	.004	1.02 (0.60–1.73)	.952
Family size (1–3 = 1)						
4–5	1.44 (1.11–1.87)	.006	1.05 (0.72–1.53)	.820	1.17 (0.67–2.07)	.580
6 and more	1.58 (1.10–2.27)	.014	0.91 (0.58–1.42)	.665	1.21 (0.60–2.45)	.600
Family history of HTN (No = 1)	2.15 (1.67–2.76)	.000	82.56 (50.57–134.79)	<.001	6.29 (2.20–17.98)	.001
Tobacco use (No = 1)			1.32 (0.95–1.85)	0.100		
First seek care (Government = 1)					0.76 (0.46–1.27)	.292
Insurance (Not insured = 1)	1.13 (0.90–1.41)	.305	1.00 (0.76–1.32)	.996	2.18 (1.39–3.41)	.001

1 indicates reference group.

AOR = adjusted odds ratio, CI = confidence interval.

## 4. Discussion

This study presents the pattern of care for DM and HTN in Bidur Municipality. The screening rates for DM and HTN observed in this study are higher compared to national data from the STEPS survey 2019 (DM: 43% vs 17%, HTN: 65% vs 56%).^[6]^ This may be because the study area is located in an urban setting, close to the capital city, which likely provides better facilities and easier access to healthcare services. However, a lower screening rate for DM compared to HTN was noted in both surveys. This discrepancy may be attributed to the simplicity and accessibility of blood pressure measurement, which is noninvasive and widely available. In contrast, DM screening typically requires invasive procedures like blood or urine testing, which may not be as accessible. To address the gap in DM screening, it is essential to expand screening programs at the primary health care level by increasing service coverage and enhancing the capacity of healthcare providers to deliver non-communicable diseases services effectively.

In this study, 19% and 26% of participants self-reported having DM and HTN, respectively. These rates are higher than those reported in the STEPS survey (DM: 2%, HTN: 12%).^[6]^ The higher rates in this study may be linked to increased screening rates in Bidur Municipality compared to the broader population surveyed in the STEPS survey.

Among participants diagnosed with DM and HTN, more than 70% reported ever receiving treatment, and over 90% of those treated adhered to their treatment regimen. These rates are higher compared to national data from the STEPS survey (adherence rate for DM and HTN: 55% and 71%, respectively).^[6]^ The higher adherence rates observed in Bidur Municipality suggest stronger continuity of care, possibly due to better healthcare service delivery or community awareness. However, barriers preventing the remaining patients from accessing or adhering to treatment need further investigation. Interventions should focus on addressing financial constraints, as DM and HTN require long-term or even lifetime medication, which may be unaffordable for economically disadvantaged populations. Additionally, many studies have reported that patients with chronic conditions may avoid initiating treatment due to concerns about long-term dependency on medication, often resorting to traditional or behavioral practices instead.^[7–9]^ Addressing these barriers through targeted awareness programs and financial support mechanisms is crucial to ensure comprehensive management of non-communicable diseases in the community.

Increasing age was found as a significant predictor for both the screening and diagnosis of DM and HTN. Age is a well-recognized risk factor for chronic diseases and is well perceived by communities, potentially leading older individuals to seek screening and diagnosis.^[10,11]^ Additionally, the perceived risk of cardiovascular events with advancing age may contribute to increased screening rates. However, while age was a predictor for current treatment in HTN, it was not for DM. This discrepancy might be explained by a perception that DM management primarily involves dietary modifications, such as reducing sugar intake, rather than medication, leading some individuals to prioritize lifestyle changes over pharmacological treatment.^[12]^

The Brahmin/Chhetri have higher screening rates compared to other ethnic groups. Brahmin/Chhetri are traditionally considered upper-class in Nepalese society, and generally have higher levels of education and better economic conditions. These factors may contribute to greater awareness regarding these chronic conditions. However, other ethnic groups had a higher burden of diagnosed cases (DM: AOR = 1.93 (1.03–3.63), HTN: 0.53 (0.39–0.72)), despite lower screening rates. Furthermore, Janajati groups showed lower odds of being on current treatment for HTN compared to Brahmin/Chhetri groups (AOR 0.51 (0.31–0.84)). These findings highlight the need for targeted awareness campaigns and community-based screening programs tailored to the needs of marginalized groups to improve early detection and treatment uptake.

A family history was found to be a strong predictor of screening, diagnosis, and treatment for both diseases. This is likely due to increased awareness and perceived risk among individuals with a positive family history of HTN or DM, therefore, seeking health care services more actively.^[13,14]^ Larger family size (more than 3 members) was also associated with higher screening rates compared to smaller families, which may be attributed to increased social support and shared healthcare-seeking behaviors within larger households.

Health insurance was a predictor for DM screening but not for HTN. This may be because blood pressure measurement is generally free of charge at many healthcare facilities, while DM screening involves blood tests, which incur a cost. Insured participants also demonstrated higher adherence to medication for both DM and HTN, although the finding was statistically significant only for HTN. This suggests that health insurance facilitates better healthcare utilization and adherence to treatment.^[15]^

Barriers to care for DM and HTN can exist on both the demand and supply sides. On the demand side, these include a lack of awareness, financial constraints, and sociocultural factors. On the supply side, challenges include inadequate diagnostic and treatment services, poor quality of care, and geographical inaccessibility.^[6,12,16]^ Addressing these barriers is essential for improving screening, treatment, and outcomes in managing DM and HTN.

Introducing innovative social health protection schemes to cover the costs of chronic disease services could ensure continuity of care without causing financial hardship. These schemes should address gaps in screening, diagnosis, and treatment, ensuring equitable access in both public and private healthcare systems. This is critical to reducing the unmet need for DM and HTN care, which includes individuals not screened, those diagnosed but untreated, and those treated but not achieving optimal control.

Although this study is backed up with a large sample size, some limitations exist. We employed a convenient sampling technique, so this study may be limited with some generalizability issue, and did not measure the blood pressure and blood sugar level of the participants to find out true prevalence and check for the optimal control.

## 5. Conclusion

DM and HTN screening rates were higher than national data in Bidur Municipality. However, disparities remain, with certain ethnic groups and individuals without health insurance exhibiting lower screening rates despite a higher disease burden. Older age and family history were key predictors of screening, diagnosis, and treatment. Targeted interventions for marginalized populations and expanding social health insurance coverage are critical to ensuring equitable access to care. These efforts will contribute to reducing the unmet need for DM and HTN management and improve long-term health outcomes.

## Acknowledgments

We are thankful to the students of the MBBS 43rd Batch of Maharajgunj Medical Campus for collecting data, and Bidur Municipality Office for providing permission to conduct this study.

## Author contributions

**Conceptualization:** Gambhir Shrestha, Deepak Raj Joshi, Pranil Man Singh Pradhan, Namrata Karki, Prem Basel, Smriti Pant, Tanweer Ahmad Mikrani, Khem Bahadur Karki.

**Data curation:** Gambhir Shrestha, Deepak Raj Joshi.

**Formal analysis:** Gambhir Shrestha, Deepak Raj Joshi, Pranil Man Singh Pradhan, Sushan Man Shrestha.

**Methodology:** Gambhir Shrestha, Deepak Raj Joshi, Pranil Man Singh Pradhan, Sushan Man Shrestha, Khem Bahadur Karki.

**Supervision:** Khem Bahadur Karki.

**Visualization:** Gambhir Shrestha.

**Writing** – **original draft:** Gambhir Shrestha, Deepak Raj Joshi, Pranil Man Singh Pradhan, Sushan Man Shrestha, Namrata Karki, Prem Basel, Smriti Pant, Tanweer Ahmad Mikrani, Khem Bahadur Karki.

**Writing** – **review & editing:** Gambhir Shrestha, Deepak Raj Joshi, Pranil Man Singh Pradhan, Khem Bahadur Karki.
